# Mindful Parenting: Perspectives on the Heart of the Matter

**DOI:** 10.1007/s12671-020-01564-7

**Published:** 2021-01-08

**Authors:** Jon Kabat-Zinn, Myla Kabat-Zinn

**Affiliations:** 1 UMass Medical School, 55 Lake Avenue North, Worcester, MA 01655 USA; 2 Northampton, USA

**Keywords:** Mindfulness, Mindful parenting, Childhood stress, Family stress, Public health, Meditation, Moment-to-moment awareness, Love

When we were writing our book, *Everyday blessings: The inner work of mindful parenting* (Kabat-Zinn & Kabat-Zinn 1997/2014), an alternative title we very much had in mind was *Mindful parenting: Nurturing our children, growing ourselves*. The “growing ourselves” element, although it was surrendered in favor of the first title and subtitle, was, is, and always will be an essential element of the entire endeavor. This theme and evidence in support of it can be found throughout this Special Issue. Mindful parenting is not a project to create “better” or “optimal” children (whatever that might mean), or to be “better” or “optimal” parents, but to embrace in moment-to-moment awareness as best we might the entire enterprise of parenting our children in a mutuality of love and discovery and not-knowing. We were saying as clearly and as invitingly as we could that such an intentional approach to family and relationality has the potential to catalyze a natural flowering of everybody’s capacity to grow into our full range of possibilities in the sheltering embrace of family—however it is defined and constrained in the actuality of our specific and singular situations.

The *nurturing* element was, is, and always will be at the center of mindful parenting as well. But how? What is skillful? What is inappropriate or overdoing things? How are we to avoid a tendency to unduly hover over children rather than accord them latitude and sovereignty? How do we approach the uniqueness of particular children, their needs, temperaments, specific challenges, and talents? If mindfulness is both a formal meditation practice of cultivating greater discernment and presence and, equally, a way of being in wise relationship to experience, how are we to understand its moment-to-moment embodiment in the face of life unfolding? How do we go about exercising our own sovereignty while honoring that of our individual children?

The magnitude of the challenge can be considerable and sometimes beyond daunting. It also varies hugely throughout life, and across families, cultures, and communities, compounded by endemic institutionalized inequalities and injustices, and multigenerational family patterns and inheritances. Parenting under any circumstances is inevitably stressful and heart-rending at times. Our vulnerability to suffering is hugely amplified. Stress and suffering are obviously shaped by many biological, social, psychological, and economic factors that can affect some families far more than others, or during some periods of time more than others.

Extended periods of stress have been shown to have toxic effects on brain growth and development in children, especially in particular windows of vulnerability (Lupien et al., 2009). Yet in a sense, all of childhood is a time of heightened vulnerability while also being a time of astonishing and mysterious growth and development, language acquisition being one among many of the most obvious and profound examples. But because of the intrinsic and extrinsic vulnerabilities of childhood, anything we as parents can do inwardly and outwardly to be aware of the stress our children may be experiencing can be helpful. We can also encourage them as they get older to practice regulating their reactions to stress for themselves, thereby minimizing its potential damage and the suffering it entrains. When all is said and done, the greatest antidote to stress in the lives of children is the certainty that they have intrinsic worth, that they are seen and known and welcomed, loved and accepted for who they are. Perhaps that is our deepest responsibility as parents.

And since we ourselves are also often chronically stressed, effective antidotes to stress that help modulate our own stress reactivity as parents become critical potential resources for us. The practice of mindfulness has been shown to attenuate stress in many different ways and in many different populations. In mindfulness-based stress reduction (MBSR), for example, we make a strong distinction between *reacting* to stress, which tends to be automatic, habitual, and therefore mindless, and *responding* to that very same stress in a way that is open, aware, and therefore more mindful. This is a practice in its own right, and one that grows and deepens over time (Kabat-Zinn, 2013, pp. 306–349).

At the same time, it is important to emphasize that it can be challenging in its own right, even stressful, to cultivate mindfulness in our lives as a way of being. It requires first of all *remembering* to open to and welcome the unwanted in critical moments as best we can, not always easy to do. If we cultivate mindfulness in non-stressful moments in everyday living, it becomes a lot easier to rely on one’s practice in the more difficult moments. As both a formal meditation practice and a way of being, cultivating mindfulness involves learning to drop into experience in ways that we might not be quite used to, and which may take some consistent effort to adopt on a regular basis. It invites a certain kind of gentle but disciplined observing and befriending of our own minds and of our own behavior unfolding in the present moment—and the simultaneous direct apprehending of how we are in relationship to it all. Simple, but not easy.

What is more, and surprising to many people, rather than trying to get to some special “meditative” state or condition, the cultivation of mindfulness involves not trying to “get” anywhere else, or to experience some kind of special mindful state (there isn’t any), but rather to fully inhabit the moment as it is. This perspective invites us to learn to metaphorically and literally take up residence in our own awareness and over time, come to trust its intrinsic insightfulness and warmth. It is about recognizing that every moment is a special moment, and an opportunity to drop into open-hearted spaciousness and discernment, no matter what is happening. That is not an ideal that we strive for, but an experience that is always available when we manage to get out of our own way for a moment, and as a consequence, be at least a little bit freer of some of the more imprisoning tendencies of our reactive thoughts and emotions.

Understood in this way, the practice of mindfulness does not require us to be different from who we are. Quite the contrary. It invites us to be fully who we are in any and every moment, to be as large and authentic as we actually are in the only moment we ever have. Isn’t this just what we want for our children as well as for ourselves? Cultivated in any and every moment, formally or informally, mindfulness thus becomes a radical act of both sanity and love. It can help us not lose our mind just when we need it most.

So any strategies and supports that can reveal and release that love in an authentic way, especially in key moments, is certainly welcome and worthy of inclusion in one’s repertoire of life skills and intentions. Embodied mindfulness is certainly one of those potential strategies and supports, as we have been suggesting.

However, it is a big ask. It bears repeating that the cultivation of mindfulness as a way of being and as a formal meditation practice is challenging, even stressful at times. As hinted at above, it is exceedingly hard to remember to be mindful in *any* moment, especially high tension moments, and then take steps to engage with some degree of awareness, clarity, equanimity, and kindness. This innate capacity we all have can only be called upon consistently, however, by cultivating mindfulness as best we can in all our moments. This takes a degree of intentionality and consistency, in other words, remembering at key moments, when we are so primed to go unconscious and forget.

This is precisely where a formal meditation practice can be an extremely valuable support, whatever form it might take. And while there is no one right way to practice mindfulness, either formally or informally, there are “wrong” or deluded ways to practice, so some degree of guidance and alignment with well-established meditative frameworks and disciplines can be important to support the entire enterprise over years and decades in an ever-changing environment. Happily, there are now more inspiring and useful resources than ever before to support parents in this domain.

Coming back to our book’s title, the blessings part is also key for parenting mindfully. It reveals something of the magnitude and gravitas of what is involved. The word *blessing* shares roots with the French, *blessure*, to wound, via its Indo-European root, *bhel*, from which come *blossom*, *bleed, blood*, and *blade.* A blessing thus involves a blossoming (and of course, a benediction) and also the inevitability of wounding (Editors of the American Heritage Dictionaries, 1996, p. 2097). Almost every parent will recognize that there is an element of truth in there being some degree of wounding braided into all the blossoming and the profound blessings we receive from our children. Thus, there is pain and potential suffering, and more so if we do not learn to recognize it and respond appropriately.

All parents feel at various times that we are at our wit’s end. The challenges can be daunting, especially in this era of an increasingly digitized, virtual, device-dominated, and algorithm-shaped “reality” that commodifies human attention and competes for it with increasingly addictive seductions. Beyond that, the present circumstances are nothing short of horrific and life-threatening for huge numbers of families, as we see on our own southern border in the USA, and with the increasing global displacement of peoples due to geo-political factors and the many mega-consequences of a rapidly warming planet. Nurturing children in a universe shaped by racial injustice, economic inequities, poverty, and stress of all kinds only compounds these challenges.

We certainly could use all the help we can get, both inwardly and outwardly. The “inwardly” is one way in which the practice element comes in, and thus, once again, the *growing ourselves* element. It is absolutely essential, and non-trivial that we as parents take care of ourselves to whatever degree possible so we can inhabit our moments most effectively, with our own interior resources intact and available to us. We start from wherever we find ourselves when mindful parenting becomes a path we might choose to adopt to parent more intentionally. It may be during a pregnancy, or when a baby is born, or at any time in one’s life and family trajectory, or even well before becoming a parent. Any and every moment is a good starting place.

We have far more support in this endeavor than in past decades. Witness, for example, this special issue, as well as the very existence and orientation of this journal, and the ever-increasing body of research on mindfulness published in so many different scientific and medical journals. Mindfulness has literally become a new field in medicine, in health care, in psychology, and in neuroscience. It is now recognized and, in many instances, encouraged and supported in mainstream work settings of all kinds, as well as in schools from pre-K-12 to university. This has been driven primarily by the scientific study of mindfulness-based programs such as MBSR (mindfulness-based stress reduction), MBCT (mindfulness-based cognitive therapy), mindfulness-based childbirth and parenting (MBCP; Bardacke, 2012), and many more that are designed to help us wake up to the full repertoire of our own embodied intelligences and to recognize those of our children and nurture them.

There is increasing evidence and momentum that mindfulness as a practice and as a way of being does facilitate “growth” on innumerable levels: physiological, epigenetic, neural networks and brain plasticity, telomeres, social cohesion, moral and ethical, etc. Now with the COVID-19 pandemic raging at the time of writing in the USA and in many other countries throughout the world, coupled with the disruption and loss it has brought to family life, to work, and to school around the world and across all age groups, and to economic life, the stress and mental health challenges ensuing from it will inevitably have major impacts on parents and children alike in the coming years and even decades. This makes mindfulness even more essential as a contributing element to public health and the healing of our families, and of our world.

For all these reasons, the present special issue on mindful parenting is most welcome and could not be appearing at a more opportune time. For completeness’ sake, we close with a working definition of mindful parenting that we proposed some time ago (see Bögels & Restifo, 2014, p. 104):

Mindful parenting is an ongoing creative process, not an endpoint. It involves intentionally bringing non-judgmental awareness, as best we can, to each moment. This includes being aware of the inner landscape of our own thoughts, emotions, and body sensations, and the outer landscape of our children, our family, our home, and the broader culture we inhabit. It is an on-going practice that can grow to include: (1) greater awareness of a child’s unique nature, feelings, and needs; (2) a greater ability to be present and listen with full attention; (3) recognizing and accepting things as they are in each moment, whether pleasant or unpleasant; (4) recognizing one’s own reactive impulses and learning to respond more appropriately and imaginatively, with greater clarity and kindness.
